# RNA-Eluting Surfaces for the Modulation of Gene Expression as A Novel Stent Concept

**DOI:** 10.3390/ph10010023

**Published:** 2017-02-10

**Authors:** Olivia Koenig, Diane Zengerle, Nadja Perle, Susanne Hossfeld, Bernd Neumann, Andreas Behring, Meltem Avci-Adali, Tobias Walker, Christian Schlensak, Hans Peter Wendel, Andrea Nolte

**Affiliations:** Department of Thoracic, Cardiac, and Vascular Surgery, University of Tuebingen, Calwerstraße 7/1, 72076 Tuebingen, Germany; olivia.koenig@gmx.de (O.K.); diane.zengerle@icloud.com (D.Z.); nadja.perle@gmx.de (N.P.); susanne.hossfeld@nexgo.de (S.H.); b.neumann.71@web.de (B.N.); behring.andi@googlemail.com (A.B.); meltem.avci.adali@gmail.com (M.A.-A.); tobias.walker@med.uni-tuebingen.de (T.W.); christian.schlensak@med.uni-tuebingen.de (C.S.); andrea.nolte-karayel@gmx.de (A.N.)

**Keywords:** atherosclerosis, drug eluting stents, PLGA films, gene delivery, local transfection, siICAM-1, gene knockdown, mRNA

## Abstract

Presently, a new era of drug-eluting stents is continuing to improve late adverse effects such as thrombosis after coronary stent implantation in atherosclerotic vessels. The application of gene expression–modulating stents releasing specific small interfering RNAs (siRNAs) or messenger RNAs (mRNAs) to the vascular wall might have the potential to improve the regeneration of the vessel wall and to inhibit adverse effects as a new promising therapeutic strategy. Different poly (lactic-*co*-glycolic acid) (PLGA) resomers for their ability as an siRNA delivery carrier against intercellular adhesion molecule (ICAM)-1 with a depot effect were tested. Biodegradability, hemocompatibility, and high cell viability were found in all PLGAs. We generated PLGA coatings with incorporated siRNA that were able to transfect EA.hy926 and human vascular endothelial cells. Transfected EA.hy926 showed significant siICAM-1 knockdown. Furthermore, co-transfection of siRNA and enhanced green fluorescent protein (eGFP) mRNA led to the expression of eGFP as well as to the siRNA transfection. Using our PLGA and siRNA multilayers, we reached high transfection efficiencies in EA.hy926 cells until day six and long-lasting transfection until day 20. Our results indicate that siRNA and mRNA nanoparticles incorporated in PLGA films have the potential for the modulation of gene expression after stent implantation to achieve accelerated regeneration of endothelial cells and to reduce the risk of restenosis.

## 1. Introduction

In 2012 more than 17.5 million people died from cardiovascular diseases (CVD), representing 31% of all global deaths. Atherosclerosis represents the most common cause for CVD and is especially represented in industrial nations [1]. The main causes of death among CVDs are the coronary artery diseases triggering stroke and heart attack. Herein, the blockage of blood flow leads to the interruption of oxygen supply, and finally the death of myocardial and brain cells, respectively. During the inflammatory process of atherosclerosis, low density lipoprotein (LDL) cholesterol causes the accumulation of lipids within the artery wall, followed by several events such as lesion initiation, plaque rupture, and thrombotic vessel occlusion [1,2,3]. Different therapeutic approaches for the treatment of coronary artery stenosis exist, e.g., bypass surgery or percutaneous transluminal coronary angioplasty (PTCA), which was performed in 1977 for the first time [4]. The method of choice is the minimally invasive introduction of a coronary stent by a balloon catheter during the PTCA. Nevertheless, the insertion of a stent harbors the risk of in-stent restenosis (ISR) and stent thrombosis and is of tremendous significance in daily clinical life [5]. Coronary stent implantation may cause atherosclerotic plaque rupture and the damage of the endothelial layer, resulting in the activation of a cascade of wound-healing mechanisms, known as neointimal hyperplasia [6,7]. Besides a cascade of various events taking place, such as medial smooth muscle cell proliferation and migration, platelet aggregation, release of growth factors, and extracellular matrix remodeling, inflammatory cell infiltration is the first response in neointimal hyperplasia [6]. After the deposition of activated platelets and fibrin on the de-endothelialized vessel wall, the recruitment and infiltration of leukocytes occurs due to inflammatory mediators and chemoattractant factors [8,9]. Therefore, endothelial cells (ECs) start with an enhanced expression of cell adhesion molecules (CAMs) such as intercellular adhesion molecule (ICAM)-1 (specific to T lymphocytes), P-selectin (specific to monocytes and neutrophils), E-selectin (specific to monocytes and granulocytes), and vascular cell adhesion molecule (VCAM)-1 (specific to monocytes) [8,10,11]. The CAMs enable the adhesion of leukocytes and subsequently the migration of the cells into the intima via diapedesis. As a consequence, the vascular smooth muscle cells (VSMCs) proliferate and migrate from the media into the neointima and a chronic inflammatory process is evoked [12,13]. Substantial progress against ISR was made with the development of drug-eluting stents (DES) replacing bare metal stents (BMS), with a reduction of ISR rates to 60%–80% [7]. Both the first-generation of DESs containing paclitaxel or sirolimus and also the second-generation DESs with zotarolimus or everolimus show adverse effects leading to delayed re-endothelialization and consequently to a prolonged antiplatelet therapy [14,15]. Nevertheless, the new DES era revealed promising efforts to eradicate these drawbacks [16]. For example, new drug-coated stents rely on the capture of endothelial progenitor cells with specific antibodies such as anti-CD34 for accelerated re-endothelialization after stent implantation [17,18,19,20]. Another promising therapeutic strategy is the coating of stents with agents at the molecular level. Gene-silencing stents containing small interfering RNA (siRNA) have the potential to inhibit inflammatory processes on the vessel wall by blocking the transcription of cytokine receptors or adhesion molecule proteins [9]. The short double-stranded 21- to 23-nucleotide-long siRNAs interfere with its complementary messenger RNA (mRNA) which is subsequently degraded [12]. This powerful and conserved self-defense mechanism in *Eukarya* has a threefold biological meaning: (1) it helps prevent infections by viral RNA, (2) it regulates and alters gene expression and (3) it controls a certain type of transposon. It is hence worthwhile pursuing this mechanism with respect to an elementary potential therapeutic strategy in humans. Therefore, siRNAs get designed and stabilized for therapeutic applications, overcoming the degradation of free siRNA by endo- and exonucleases in blood, in serum, and in living cells [13,14]. The liposome or lipid protection strategy is common for in vitro and in vivo siRNA transfection. Cationic lipids such as Lipofectamine^®^ 2000 are able to form nanoparticles (NPs) with siRNA as well as with mRNA, another transient gene delivery approach, which has gained increasing interest in the treatment of several diseases [15,16]. The mRNA delivered into the cell uses the cell’s own translational machinery to produce the protein it encodes. Recently, we showed the potency of a CD39 mRNA coating in reducing complications after stent angioplasty [17]. The pivotal point in designing a drug delivery system is the choice of the material where the NPs can be embedded. Both biocompatibility and hemocompatibility are equally important to ensure not only cell proliferation and viability but also a possible therapeutic application. Additionally, the release of drugs with long-term effects, controlled delivery and efficacy is of no lesser importance. One of the most popular Food and Drug Administration (FDA)- and European Medicines Agency (EMA)-approved biomaterials in drug delivery carrier systems is poly (lactic-*co*-glycolic acid) (PLGA), a copolymer of poly lactic acid (PLA) and poly glycolic acid (PGA) [21,22]. During the hydrolysis of PLGA, these two monomers emerge again with the metabolic products carbon dioxide and water. PLGA seems to provoke less systemic cytotoxicity, considering PLA and PGA naturally form in the human body via the Krebs cycle [21,22,23]. However, the production of acids upon polymer degradation is seen as a disadvantage of PLGA [24]. The degradation of commercially available PLGA is determined by the physico-chemical properties of the polymers such as the molecular weight, end groups (capped or uncapped) and PLA:PGA ratio, and it is degraded slowly over months to years in the body [25,26,27]. With a higher content of PGA in PLGA, the degradation time is increased, except for 50:50 (PLA:PGA) having the fastest degradation of one to two months (PLGA 75:25, four to five months; PLGA 85:15, five to six months) [28,29]. Therefore, PLGA is a promising biodegradable polymer for coating cardiovascular stents as a drug carrier in either thin coatings or PLGA NPs. Commercially available DESs such as Nevo™ (Johnson & Johnson) and Supralimus™ (Sahajanand Medical) consist of PLGA as a coating material for cobalt-chromium or stainless steel with sirolimus as a drug to prevent ISR [30]. Furthermore, a study by Klugherz et al. demonstrated the importance and effectiveness of plasmid DNA-loaded PLGA NPs by successfully transfecting pig arteries in vivo for the first time [31]. In 2010, Brito et al. clarified the long-term effect of PLGA in a rabbit iliac artery restenosis model with endothelial nitric oxide synthase (eNOS) expressing plasmid DNA lipoplexes, while restenosis was significantly reduced [32].

Our study deals with the development of a biodegradable coating consisting of PLGA that releases RNAs over a distinct period. Different PLGA derivatives are used to immobilize Lipofectamine^®^ 2000–encapsulated siRNA against ICAM-1 and reporter mRNA (eGFP). First, PLGA coatings were tested for pH value changes during incubation in media. Afterwards, the cell viability, transfection efficiency, and knockdown in EA.hy926 cells and human vascular endothelial cells (hVECs) were analyzed. Furthermore, the release duration of PLGA coatings and co-transfection of EA.hy926 cells with eGFPmRNA and siRNA were determined. We tested the immune response of immortalized and primary endothelial cells as well as ICAM-1 expression by quantitative RT-PCR after incubation on PLGA coatings. Additionally, the cleavage site of ICAM-1 mRNA was demonstrated by 5’-RLM-RACE-PCR. The hemocompatibility of PLGA coatings was analyzed with regard to later medical applications.

## 2. Results

### 2.1. Stability of the pH Value

The presence of an aqueous solution is responsible for the degradation of PLGA. In this case, hydrolysis provokes the biodegradation of esterase linkage into d,l-Lactic acid and glycolic acid. Consequently, the pH changes in a different manner when different PLGA monomer ratios of lactic acid and glycolic acid are used [33]. We decided to use cell culture medium instead of a simple saline solution to correspond more to in vivo conditions.

**Figure 1:**
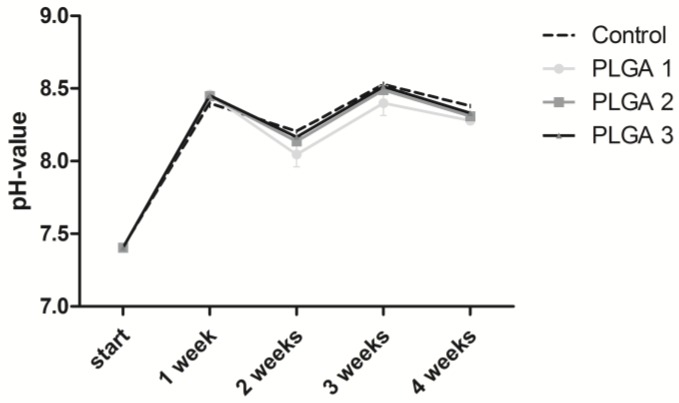
Change of pH values in cell culture media during incubation with PLGA 1-, 2-, and 3-coated coverslips. Slides were incubated in media for one, two, three, and four weeks. The supernatants were used to measure the pH values. An uncoated coverslip served as a control. The analysis was done with two equally coated coverslips of each PLGA and two measurements for each supernatant.

The pH value showed an increase in the first week for all PLGAs analyzed (Figure 1). Lower pH values were observed for PLGA 1 after two, three, and four weeks and for PLGA 2 after two and four weeks compared to PLGA 3.

### 2.2. Influence of PLGA on Cell Viability

**Figure 2:**
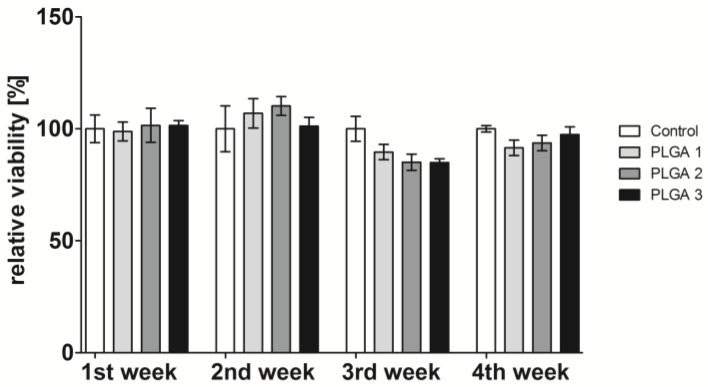
Relative viability of EA.hy926 cells analyzed by MTT assay after incubation with supernatant of incubated PLGA 1–, 2– or 3–coated coverslips. Therefore, 100,000 EA.hy926 cells were seeded in one well of a 24-well plate and cultivated with the supernatant of the respective cultivated PLGA slides for 48 h. For the control group, medium was incubated with coverslips and supernatant added to the cells. The control was set to 100%, each bar represents the mean ± standard error (SEM) of *n* = 1.

The influence of PLGA degradation products on cell viability was analyzed after 48 h cultivation. In advance, PLGA-coated slides were incubated with medium for different time points. The cells were grown using these supernatants. The cell viability was measured by MTT assay (Figure 2) and CASY^®^ (Supplementary Figure S1). Both techniques showed similar cell viability results for the whole observation period of four weeks. No changes in the cell viability occurred after incubation with one- and two-week-old supernatants of the different PLGAs. However, a slight but not significant decrease of viability was recognized when cells were cultivated with three-week-old supernatants. Herein, PLGA 2 and 3 reduced the viability to 85%, while PLGA 1 caused a slight reduction to 90% viability. The four-week-old supernatants of PLGA 2 and 3 reduced the cell viability to a lesser extent.

### 2.3. Hemocompatibility

**Figure 3:**
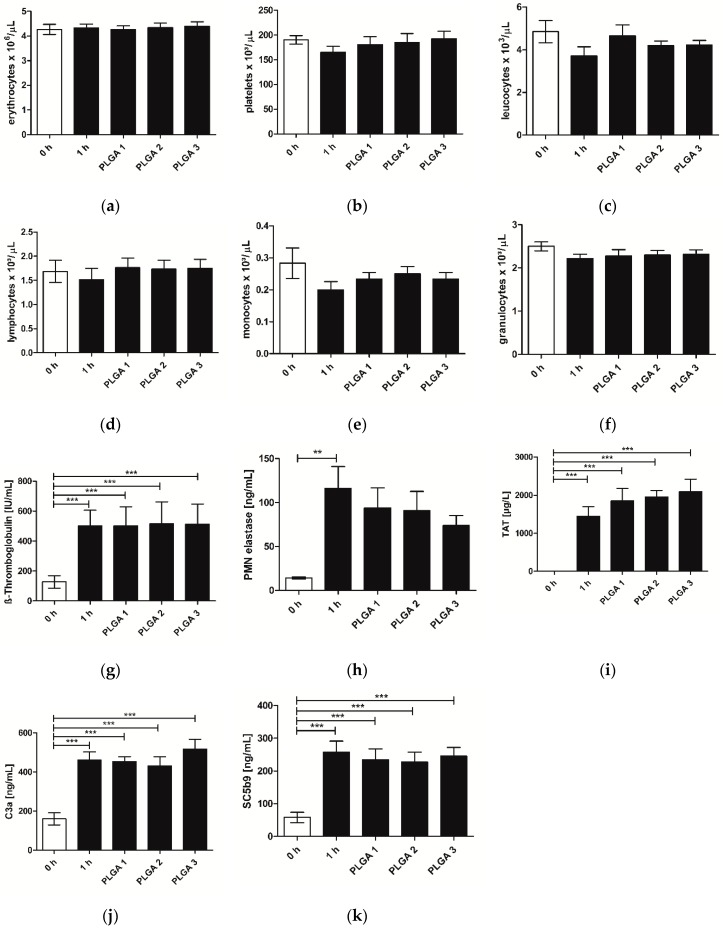
Hemocompatibility of PLGA 1–3–coated slides. Slides were incubated with fresh human blood at 37 °C for 1 h under gentle shaking. The 0 h control was fresh blood without incubation and served as a baseline. Different blood cells, inflammatory and thrombogenic parameters were determined by a cell counter (**a**) erythrocytes × 10^3^ µL^−1^; (**b**) platelets × 10^3^ µL^−1^; (**c**) leukocytes × 10^3^ µL^−1^; (d) lymphocytes × 10^3^ µL^−1^; (**e**) monocytes × 10^3^ µL^−1^; (**f**) granulocytes × 10^3^ µL^−1^ or appropriate ELISAs (**g**) β-Thromboglobulin (IU·mL^−1^); (**h**) PMN elastase (ng·mL^−1^); (**i**) TAT (µg·L^−1^); (**j**) C3a (ng·mL^−1^); (**k**) SC5b9 (ng·mL^−1^), respectively. The comparison between the uncoated slides and the PLGA-coated slides shed light on the hemocompatibility of the polymer. Each bar represents the mean ± standard error (SEM) of *n* = 6. ** indicates statistical significance at a level of *p* < 0.01; *** indicates statistical significance at a level of *p* < 0.001.

With regard to the requirements of hemocompatibility tests for medical devices, different parameters concerning white and red blood cells, platelets, coagulation, and parameters of the immune system were analyzed. None of the different blood cells analyzed showed a significant reduction of their cell number in comparison to the controls (0 h and 1 h). However, it has to be mentioned that the cell numbers of platelets (Figure 3b), leukocytes (Figure 3c), lymphocytes (Figure 3d), monocytes (Figure 3e) and granulocytes (Figure 3f) were reduced after 1 h incubation in the control, whereby incubation with PLGA resulted in a similar cell number compared to the 0 h control (Figure 3a–e). Assessing the blood parameters β-Thromboglobulin, Thrombin-Antithrombin III-complex (TAT), and polymorphnuclear granulocyte (PMN)-elastase as well as the values of the complement system C3a and SC5b9, a significant increase appeared between the 0 h control and samples that were incubated for 1 h (Figure 3g–k). There was no difference between blood incubated with or without PLGA for 1 h, suggesting that no activation of the complement system as well as no activation of platelets occurred. Additionally, coated slides provoked a slight decrease of the PMN-elastase in comparison to the 1 h control, and therefore a decrease of the inflammatory reaction (Figure 3h). It has to be mentioned that PLGAs triggered a slight increase of the coagulation activity determined by TAT expression; however, this was not significant (Figure 3i).

### 2.4. Immune Response of hVECs to Different PLGAs

**Figure 4:**
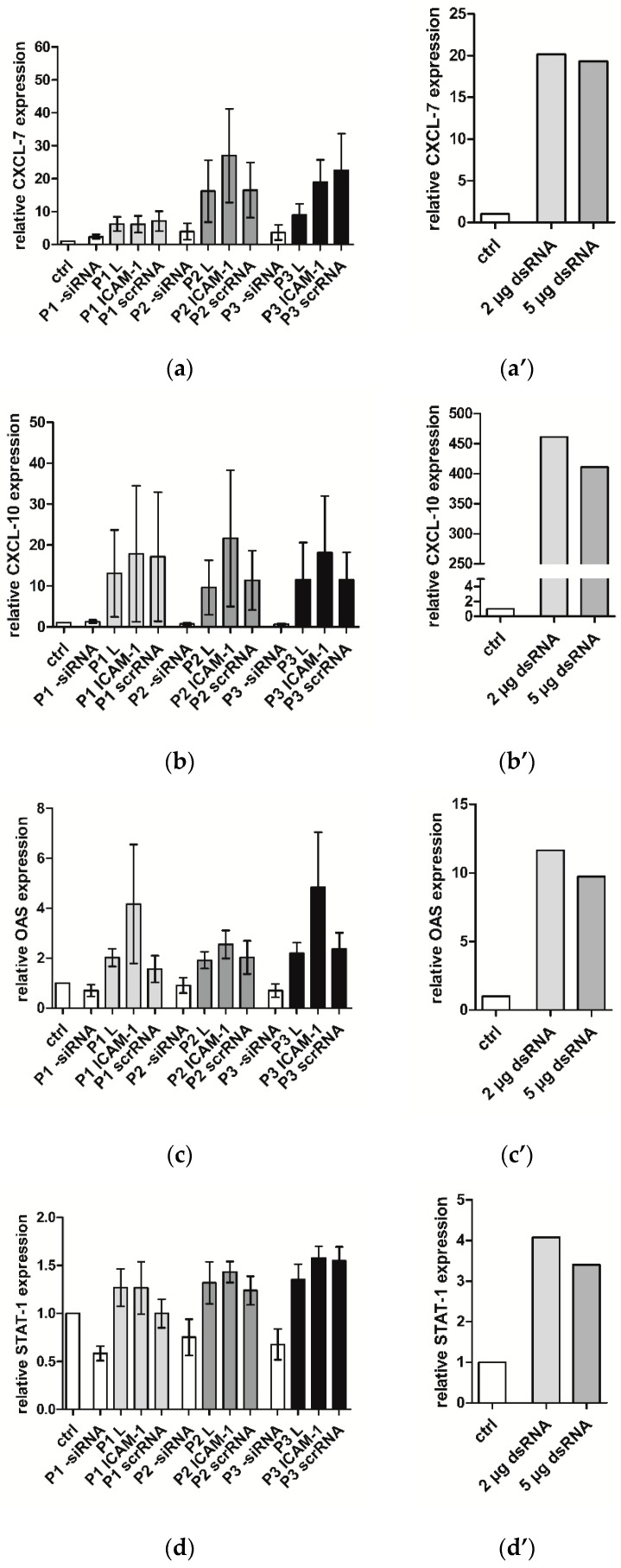
Immune response of hVECs analyzed by qRT-PCR after incubation with siICAM-1, scrRNA or Lipofectamine^®^ 2000 alone, PLGA 1–3 coatings and PLGA 1-3 alone. After 24 h incubation of coated slides with hVECs, RNA was isolated for qRT-PCR. The relative expression of (**a**) CXCL-7, (**b**) CXCL-10, (**c**) OAS, and (**d**) STAT-1 was compared to untreated cells. Another assay was the transfection of hVECs with dsRNA (2 and 5 µg): (**a’**) relative expression of CXCL-7, (**b’**) relative expression of CXCL-10, (**c’**) relative expression of OAS, and (**d’**) relative expression of STAT-1. The expression of untreated cells was set to one. Each bar represents the mean ± standard error (SEM) of *n* = 4. The dsRNA was tested only once, *n* = 1. *p* = PLGA.

Biomaterials and external molecules such as RNAs may trigger an immune response in cells which is not desirable, especially for medical devices. Therefore, the expression of different inflammatory markers such as CXCL-7, CXCL-10, OAS, and STAT-1 was determined after the incubation of hVECs with PLGA 1–3 coatings in combination with Lipofectamine^®^ 2000, siICAM-1 and control nonsense siRNA (scrRNA), or the transfection of poly (IC) double-stranded RNA (dsRNA) with Lipofectamine^®^ 2000. The dsRNA induced the mRNA expression of CXCL-10, OAS, and STAT-1 mRNA to a greater extent than siICAM-1 or scrRNA PLGA coatings (Figure 4). When looking at the results in detail, all three PLGAs in combination with Lipofectamine^®^ 2000 alone or siICAM-1 or scrRNA provoked the same slight increase in the mRNA level. However, PLGA without transfection solution and transfection complexes showed almost no augmentation in the expression level (Figure 4a) or even a lower level than the control (Figure 4b–d). The addition of Lipofectamine^®^ 2000 alone to the PLGAs caused a remarkable increase of the inflammatory markers, which was further intensified by siICAM-1 or scrRNA.

### 2.5. Short-Term Uptake of siRNA AF488

**Figure 5:**
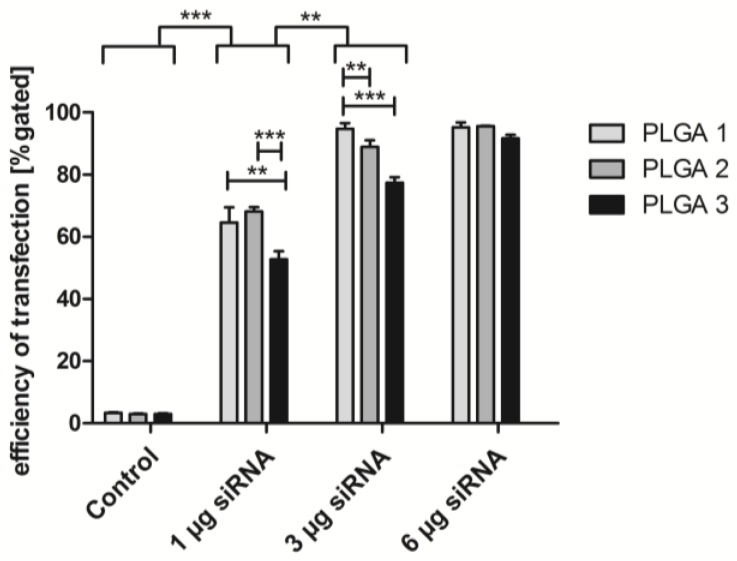
Uptake of siRNA AF 488 by PLGA coatings in EA.hy926. Different amounts of siRNA were combined with PLGA 1–3 and tested for transfection efficiency. Cells were incubated 24 h with respective coated glass slide and afterwards analyzed by flow cytometry. PLGA-coated slides without siRNA served as a control. Statistical analysis was prepared by two-way ANOVA. Each bar represents the mean ± standard error of *n* = 3. ** indicates statistical significance at a level of *p* < 0.01; *** indicates statistical significance at a level of *p* < 0.001

The three different PLGAs were tested for their ability to release siRNA complexes for transfecting EA.hy926. The transfection efficiency was determined by three siRNA AlexaFluor (AF) 488 amounts: 1, 3, and 6 µg. The cultivation of EA.hy926 with the functional polymer coating yielded the following results (Figure 5). The increase of the siRNA amount correlated with the transfection efficiency, independently of the kind of PLGA. For two-way ANOVA, we grouped the samples of different amounts of siRNA and the respective PLGAs. Comparing the group of 1 µg and 3 µg siRNA, we recognized that significantly higher uptake rates were reached with 3 µg. The application of 6 µg siRNA could not cause a significantly higher transfection efficiency anymore. 96% positive cells were observed with PLGA 2 and 95% positive cells with PLGA 1. However, PLGA 3 mediated the lowest uptake rate of 92% in comparison with the other PLGA coatings. Hence, in subsequent experiments, 3 µg siRNA was introduced in combination with PLGA 1–3. Furthermore, PLGA 1–3 showed significant distinctions in siRNA complex uptake using 1 µg and 3 µg siRNA. Herein, PLGA 3 yielded the lowest uptake in EA.hy926 with 53% (1 µg siRNA) and with 77% using 3 µg siRNA. The transfection efficiency by PLGA 1 and 2 was significantly higher except between PLGA 2 and 3 with 3 µg siRNA. The comparison of PLGA 1–3 showed that PLGA 1 was significantly more successful for the transfection efficiency than PLGA 2 and 3.

### 2.6. ICAM-1 Knockdown and mRNA Levels of EA.hy926 and hVECs

**Figure 6:**
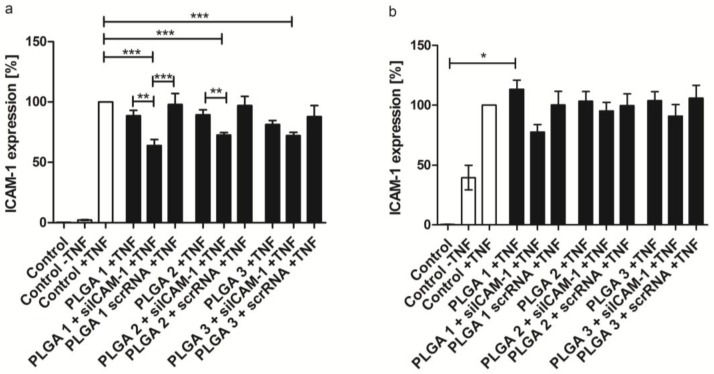
Gene knockdown of ICAM-1 after 48 h transfection by PLGA-coated slides examined by flow cytometry. (**a**) Expression of ICAM-1 in EA.hy926; (**b**) Expression of ICAM-1 in hVECs. EA.hy926 and hVECs were seeded one day before transfection with PLGA 1–3–coated glass slides in combination with either siICAM-1 or scrRNA or without additives, which served as a control. ICAM-1 expression was calculated after setting the TNF-α control to 100%. Each bar represents the mean ± standard error of *n* = 5. * Statistical significance *p* < 0.05; ** statistical significance *p* < 0.01; *** statistical significance *p* <0.001.

The transfection of EA.hy926 and hVECs was investigated with the three different PLGA coatings including 3 µg siICAM-1 and scrRNA complexes, respectively. The protein expression of the adhesion molecule ICAM-1 expressed by EA.hy926 and hVECs was analyzed by flow cytometry (Figure 6) as well as its mRNA levels analyzed by qRT-PCR (Supplementary Figure S2). All three PLGA coatings were able to reduce the ICAM-1 protein expression significantly in EA.hy926 compared to the control group, activated by TNF-α. The highest knockdown of 36% was reached with PLGA 1 combined with siICAM-1. This reduction was also significant compared to scrRNA and the TNF-α control. Similar significant reductions were achieved with PLGA 2 and 3 compared to the TNF-α control. Furthermore, a significant reduction was also mediated by siICAM-1 PLGA 2 compared to the TNF-α control. Additionally, these results were paralleled by the mRNA level of ICAM-1 (Supplementary Figure S2). The knockdown experiment was also carried out with hVECs and a reduction of the ICAM-1 expression of 22% (PLGA 1), 10% (PLGA 3), and 5% (PLGA 2) could be achieved. PLGA alone and the scrRNA PLGA coating with TNF-α stimulation revealed no reduction of the examined protein. Nevertheless, comparing the results of EA.hy926 and hVECs, significant gene knockdown could not be detected with primary cells.

### 2.7. Specific mRNA Degradation Analyzed by 5′-RLM-RACE-PCR

**Figure 7:**
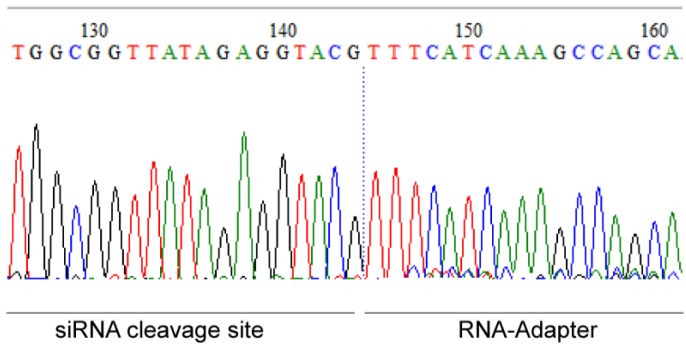
Sequence of 5′-RLM-RACE-PCR product (Inner PCR with Inner Primer and RACE1) of PLGA-ICAM-1 siRNA–treated EA.hy926 cells. The sequence shows the siRNA cleavage site of the ICAM-1 mRNA at bp 1818 (126–144) and the RNA Adapter sequence (145–161).

The analysis of specific mRNA degradation products is very important to distinguish between the desired specific RNA interference (RNAi) mechanism and the undesired effects that are mediated by the biomaterial, transfection reagent, etc. These undesired effects may also lead to a knockdown of the targeted ICAM-1 mRNA. With the usage of 5′-RLM-RACE-PCR we could prove that the RNAi mechanism occurred in EA.hy926 cells at the siRNA cleavage site of the mRNA (Figure 7). Our analysis revealed that the mRNA transcript was cleaved at bp 1818 as anticipated by us. PLGA-scrRNA or PLGAs without siRNA did not show specific ICAM-1 mRNA cleavage products (data not shown).

### 2.8. Long-Term Release of siRNA Complexes

**Figure 8:**
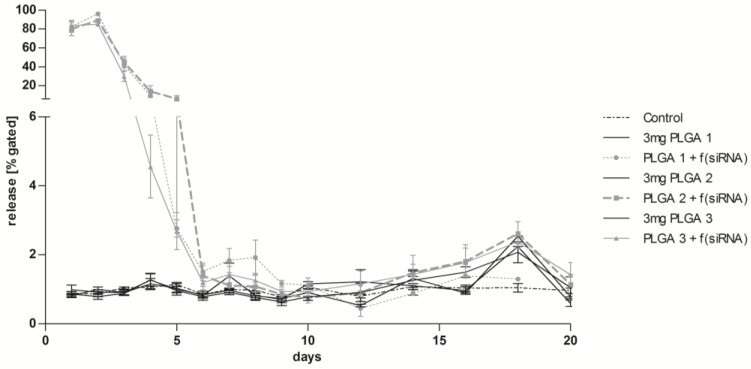
Long-term release of siRNA AF 488 complexes and the conditional transfection efficiency in EA.hy926. Glass slides were coated four times with alternating layers of 0.5 mg PLGA and 3 µg siRNA AF 488 with a capping layer of 1 mg PLGA. In the first 10 days, slides were transferred to new confluent cells every day. From day 11, slides were changed every second day. As control samples, cells without a slide and slides coated with 3 mg PLGA only were used. Uptake of siRNA was measured by flow cytometry. f(siRNA) = fluorescence-labeled siRNA. Statistical analysis was performed by an outlier test (alpha = 0.05) and test for normal distribution before the ANOVA. Each bar represents the mean ± standard error of *n* = 4.

With respect to the requirements of long-term release coatings for medical devices, we developed and analyzed a multilayer buildup. During the first two days of transfection by PLGA-coated glass slides with siRNA AF 488, a very high transfection efficiency of at least 80% on day one and 85% on day two could be achieved (Figure 8). The highest value was reached with PLGA 1 (96%). From the third day on, the uptake of fluorescent siRNA decreased continuously until day six, where a baseline was reached with 1%–2% transfection efficiency. PLGA 1 showed a slight increase of transfection efficiency from day seven until day nine, which was not significant compared to PLGA 2 and 3. Release of siRNA AF 488 was tested until day 20. The transfection efficiency was slightly but continuously increased by PLGA 2 and 3 from day 12 to day 18. PLGA control slides showed baseline values except for day 18.

### 2.9. Co-Transfection of eGFPmRNA and siRNA AF 555 in EA.hy926

Gene silencing by RNAi is not the only way to alter protein expression. Also, the transfection of mRNA is capable of changing the protein expression pathway. In our approach we wanted to ascertain if the PLGA coating is capable of releasing and transfecting both transfection complexes with eGFPmRNA and siRNA AF 555 simultaneously. Different combinations of PLGA 1 (which was the most efficient PLGA in the former experiments) and RNAs were tested. The co-transfection achieved 11% transfected cells that were positive for both eGFPmRNA and siRNA AF 555 (Table 1). Furthermore, the values of eGFP expression and cells positive for AF 555 separately showed 7.90% positive cells for mRNA and 6.25% positive cells for siRNA when both siRNA and mRNA were immobilized in the coating. As a control, coatings with only one complexed RNA were tested for transfection efficiency. Regarding the combination of PLGA and siRNA, 26% transfected cells could be achieved, while the PLGA/mRNA coating also provoked 11% positive cells. Table 1 shows an overview of the transfection efficiency values. The analysis of the mRNA and siRNA transfection was also done by fluorescence microscopy (Supplementary Figure S3), with which it was visible that some cells efficiently expressed eGFP or/and were transfected with siAF555 simultaneously.

## 3. Discussion

The placement of stents by PTCA is a common treatment of narrowed arteries in atherosclerosis. Nowadays, DES releasing paclitaxel or sirolimus is the treatment of choice to circumvent restenosis, although adverse effects such as late ISR may occur. Huge efforts are being made to improve the re-endothelialization of stented arteries and new delivery systems are constantly being developed. Some of the new inventions use biodegradable biomaterials for stent coating, to incorporate pharmacological agents in a depot for controlled and sustained release. However, such a biomaterial has to be carefully selected regarding its biodegradability without toxic degradation products, stabilization of the incorporated drug, controlled drug release, bioresorbability, hemocompatibility, and inappropriate immune response. Peng et al. revealed in a porcine coronary model that PLGA-coated stents showed a long-term biocompatibility of three months and the degradation products led to fewer reactions compared to BMS [34]. In this respect, we used the biocompatible and biodegradable FDA- and EMA-approved biomaterial PLGA with different lactide:glycolide ratios, different molecular weights, and either end-capped or non-end-capped polymers. We determined the biocompatibility of PLGA 1, 2, and 3 by looking at the pH value shift and the cell viability over four weeks. Hereby, we observed a pH value rise from the beginning until the first week that might be due to the pH augmentation of the cell culture medium in which the coatings were incubated. It is well known that the pH of a cell culture medium shifts towards basic conditions after refrigerator storage. Additionally, we assume that the cell culture medium was capable of buffering the pH value, resulting in no decrease of the pH due to the hydrolysis of PLGA. The cell viability showed no reduction after the first two weeks, indicating that the increase of the pH value from 7.4 to 8.4 has no influence (Figure 1). Furthermore, cell viability was only slightly but not significantly decreased after the third and fourth weeks. This leads to the conclusion that PLGA does not cause cytotoxicity, since the reduction of the cell viability was less than 30% (determined value according to EN ISO 10993-5) (Figure 2). 

The comparison of the three PLGAs regarding the pH value is in line with the literature stating that free carboxylic acid end groups of PLGA 1 and a low molecular weight are responsible for faster degradation as compared to PLGA 2 and 3 with capped carboxyl end groups [26,27]. With free carboxylic acid end groups, the polymer is becoming more hydrophilic, leading to increased water uptake and consequently to hydrolysis. Hence, the carboxylic acid end groups explain the decreased pH value of PLGA 1 from week two until week four compared with the other PLGAs and the control (Figure 1).

The excellent hemocompatibility of PLGA is essential for future medical device applications. In contrast to the 1 h control, all three PLGA samples caused no reduction in the number of platelets, leukocytes, lymphocytes, monocytes, and granulocytes after incubation (Figure 3). A reduction of their numbers would indicate the adherence to the PLGA surface, which was not observed. Therefore, we conclude that there is no adverse adhesion of blood cells onto the polymer coatings. Furthermore, the hemocompatibility of PLGAs was verified by the unchanged parameters ß-Thromboglobulin, TAT, PMN-elastase and complement C3a and SC5b9 after incubation. This confirmed that no activation of the complement system as well as no activation of platelets occurred, reducing the risk of thrombus formation after PTCA.

The expression of inflammatory and interferone response markers was not increased by PLGA. When Lipofectamine was added, the markers increased. Lipofectamine is a cationic lipid which forms nanoparticles that are taken up by the cell. In a previous study we could also observe the activation of inflammatory markers when using Lipofectamine [35]. The addition of siRNAs to this experimental setup led to a still-higher inflammatory marker increase. 

Although it is thought/intended that siRNAs are specific and only interact with the RNA-induced silencing complex and do not lead to any increase in inflammatory markers, we can assume that siRNAs can facilitate a certain activation of inflammatory markers. This effect belongs to the dsRNA of 21 nucleotides or longer that can bind directly to Toll-like receptor 3. This results in receptor dimerization and activation of an intracellular pathway, leading to the expression of inflammatory markers. It must be noted, however, that the use of a long dsRNA leads to a much higher activation of the inflammatory markers.

In our study, we generated drug delivery particles consisting of ICAM-1 siRNA and Lipofectamine and embedded them in thin PLGA films. The therapeutic approach of RNAi holds a lot of promise in combating CVD by preventing the inflammatory process. Several studies provided substantial evidence that the knockdown of CAMs such as ICAM-1, VCAM-1, and E-selectin is an excellent tool to prevent the inflammatory process by leukocyte infiltration within different cell types of the artery wall [36,37,38,39,40,41]. Based on this available evidence, we successfully transfected EA.hy926 cells with siRNA AF 488 released from PLGA 1–3 thin films. Even 3 µg of siRNA was sufficient to achieve a transfection efficiency of 95% which could not be significantly augmented with a higher amount of siRNA. Therefore, we conclude that cells are saturated at 3 µg siRNA. However, only PLGA 1 was capable of reaching such an efficiency, convincing us again that PLGA 1 is the fastest in degradation. In our previous studies with different materials, it turned out that fast-degrading materials often show the highest transfection efficiency [42,43]. Additionally, a DES for long-term release of siRNA NPs from PLGA multilayer coatings was conducted and confirmed by their uptake in EA.hy926 cells for 20 days. The rationale of generating several alternating layers is: (1) decelerating the NPs’ release due to several PLGA films that have to be passed and (2) the immobilization of mRNA and siRNA into different layers to yield the gradual release of both. Within the first two days, we demonstrated a very high uptake of siRNA NPs, demonstrating a burst release mainly by diffusion of the water-soluble NPs. However, from days three to six, cells showed a decreasing uptake of siRNA NPs, which indicates the beginning of the slow degradation of PLGA films (Figure 8). Additionally, a progressively low uptake of siRNA NPs was seen until day 20, indicating that a sustained release of NPs from PLGA multilayers is guaranteed. The in vitro experimental setup, relocating the coated slides on new EA.hy926 cells until day 10 for every day and until the end of the experiment for every second day, simulated a very high turnover of endothelial cells in vivo. Considering that the endothelium is only renewed once after stent implantation, not every (second) day, the transfection duration and efficiency would be significantly higher in vivo compared to our results. 

Several PLGA degradation mechanisms are described in the literature which might lead to the release of our embedded siRNA NPs: (1) diffusion through the polymer barrier, (2) erosion (physical) and degradation (chemical) of the polymer material or (3) a combination of diffusion and erosion/degradation [44,45]. An enzymatic degradation of PLGA besides the proven hydrolysis is still debated [46,47]. Therefore, we presume that in our case, siRNA NPs were released by diffusion (for the first two days) through the polymer, by degradation (hydrolysis), and by erosion.

A further advantage of our multilayered coating approach is the possibility of co-transfecting eGFPmRNA and siRNA AF 555 for long-term release. This is, to our knowledge, the first description of this mechanism and its feasibility has been proven. The simultaneous transfection of EA.hy926 cells with mRNA and siRNA convinced us of the novel concept to build up several layers with different RNAs (Table 2). Our results indicate the possibility that two contrary mechanisms in the protein expression machinery occur concurrently in EA.hy926: (1) gene knockdown by siRNA transfection and (2) protein expression by mRNA transfection.

The effectiveness of PLGA carrier-mediated transfection was extensively confirmed by the gene knockdown of adhesion molecule ICAM-1 in EA.hy926 cells and hVECs. The highest knockdown was observed with the combination of PLGA 1 and siICAM-1 in the cell line and primary cells, leading to the assumption that faster degradation of PLGA leads to faster release of siICAM-1 and consequently to a significant (EA.hy926) ICAM-1 knockdown (Figure 6a). Additionally, we confirmed decreased ICAM-1 expression with PLGA 2 and 3 by flow cytometry. We thus suppose that PLGA 1 is the most suitable coating for significant ICAM-1 gene silencing or for co-transfection. The results of the qRT-PCR underline our assumptions, where the lowest ICAM-1 mRNA level with 53% (in EA.hy926 cells) was achieved with the PLGA 1 coating (Supplementary Figure S2). To assure that our siICAM-1 was able to specifically degrade target mRNA, 5´-RLM-RACE-PCR was performed. The correct cleavage site seen with ICAM-1, but not with the scrRNA coating or PLGA alone, confirmed the functionality. In conclusion, siICAM-1 in combination with PLGA 1 was fully functional and the side effects of PLGA or scrRNA could be excluded.

Comparing the ICAM-1 expressions of EA.hy926 cells and hVECs, our data are in accordance with the literature that efficient gene transfer is more difficult to manage in primary cells than in cell lines [48,49]. In our view, too few siRNA NPs reached the cell cytoplasm for effective gene silencing or an unwanted effect of Lipofectamine was responsible for the lower knockdown in primary cells. A different transfection reagent, higher amounts of the transfection reagent or increased RNA amounts could achieve improvements of the transfection. We could show in our previous work that other transfection reagents might be more suitable for the transfection of primary cells [35]. Moreover, using different transfection reagents for siRNA and mRNA would equally be possible as they can be incorporated into different layers.

## 4. Materials and Methods

### 4.1. Chemicals for PLGA Coating

Three different PLGAs were purchased from Sigma-Aldrich (Steinheim, Germany): PLGA (1) Resomer^®^ RG 752 H, 75:25, acid terminated, 4,000–15,000 Da; PLGA (2) PLGA, 85:15, ester terminated, 50,000–75,000 DA; PLGA (3) Resomer^®^ RG 756 S, 75:25, ester terminated, 76,000–115,000 Da. Subsequently, PLGAs are named with PLGA 1, PLGA 2, and PLGA 3. Ethyl acetate from Sigma-Aldrich (Steinheim, Germany) was used for preparing PLGA solutions.

### 4.2. SiRNAs

The following siRNA was used for gene knockdown: intercellular adhesion molecule (ICAM)-1 hum with sense strand 5′-GCC UCA GCA CGU ACC-UCU ATT-3′’, antisense 5′-UAG AGG UAC GUG CUG AAG CTT-3′. Alexa Fluor 488 labeled E-selectin siRNA was applied to test the transfection efficiency: sense strand 5′-UUG AGU GGU GCA UUC AAC CTT-3′, antisense 5′-GGU UGA AUG CAC CAC UCA ATT-3′ (both from Eurofins MWG Operon, Ebersberg, Germany), and scrRNA (Qiagen, Hilden, Germany) without labeling and with AF 488 and AF 555 labeling, respectively. Qiagen does not provide the sequence of their nonsilencing siRNAs but ensure that they have no homology to any known mammalian gene. This nonsilencing siRNA is validated by using Affymetrix GeneChip arrays and a variety of cell-based assays and shown to ensure minimal nonspecific effects on gene expression and phenotype. Additionally, scrRNA AF488 from Qiagen was used for long-term release experiment.

### 4.3. Synthesis of eGFPmRNA

The production of modified mRNA was performed as described by Avci-Adali et al. [50]. Coding DNA sequence (CDS) with known flanking sequences was amplified by PCR using specific primers. PCR product was then purified and the quality of the generated DNA was determined. Using the in vitro transcription (IVT) process, mRNA was generated from the DNA product. Subsequently, the product was purified and treated with phosphatase to remove 5′-triphosphates. After the additional purification and quality control of generated mRNA, transfection experiments were performed.

### 4.4. Substrate for PLGA Coatings

PLGA films were build-up on glass slides from Paul Marienfeld GmbH (Lauda-Königshofen, Germany). The dimension of the slides was 10 × 10 × 1 mm. They were purified by ultrasonication (Bandelin RK 100H Sonorex, Bandelin electronic, Germany) with 2% Hellmanex solution from Hellma (Müllheim, Germany) before rinsing with ddH_2_O. Air-dried slides were sterilized by steam sterilization for 20 min at 121 °C (systec dx-23, systec GmbH, Linden, Germany) prior coating.

### 4.5. Build-Up of PLGA/RNA Coatings

PLGA 1, 2, and 3 were dissolved in ethyl acetate with a concentration of 1 µg/µL. For every experiment, PLGA solutions were daily prepared. The complexation of Lipofectamine^®^ 2000 and siRNA was obtained by diluting them in medium (DMEM) for 30 min at RT. In this connection, Lipofectamine amounts varied in relation to increasing siRNA amounts: 1 µg siRNA and 1 µL Lipofectamine, 3 µg and 2 µL, and 6 µg and 4 µL. PLGA/Lipofectamine coatings without siRNA served as a control. PLGA and Lipofectamine/siRNA complexes were mixed with a 1:1 ratio and 100 µL of coating solution consisting of Lipofectamine complexed siRNA and PLGA were pipetted onto the glass slides.

For analysis of the long-term transfection efficiency, a multilayer build-up of PLGA and Lipofectamine/siRNA particles was deposited onto glass slides. Then 3 µg AllStars Neg. siRNA AF 488 (Qiagen, Hilden, Germany) was complexed with Lipofectamine. The multilayered coatings were alternately dried with 500 µg PLGA and 3 µg siRNA complexes for four times, resulting in 2 mg PLGA and 12 µg siRNA for one glass slide. To prevent fast degradation of layers, a capping layer of 1 mg PLGA was deposited on top. For the control, slides were coated with a single layer of 3 mg PLGA.

The co-transfection analysis was done with PLGA 1, eGFPmRNA, and siRNA AF 555 (Qiagen). Nunc™ Thermanox™ Coverslips, dia. 13 mm (Thermo Fisher Scientific, Waltham, MA, USA) were used for coating. Therefore, PLGA solution (50 µg in 50 µL/coverslip) was mixed with a transfection mix consisting of 10 µg eGFPmRNA, 3 µg siRNA and 2 µL Lipofectamine^®^ 2000. Afterwards the transfection solution was pipetted and dried on coverslips for one night. Then, the slides were laid down on confluent EA.hy926 cells for 48 h in 12-wells and later analyzed by FACS or fluorescence microscopy. 

### 4.6. Cultivation of EA.hy926 and Human Primary Endothelial Cells (hVECs)

The human umbilical vein cell line EA.hy926 (LGC Standards GmbH, Wesel, Germany) was used for cell experiments. Cells were cultured in Dulbecco’s Modified Eagle’s Medium (DMEM) high glucose containing 10% fetal bovine serum (FBS), 1% Penicillin/Streptomycin, and 1% l-glutamine.

Human primary endothelial cells (hVECs) were isolated from saphenous vein specimens obtained from patients undergoing elective coronary artery bypass grafting (CABG). The patients’ statement of agreement and the permit of the Clinical Ethics Committee of the University of Tuebingen authorized the handling. ECs were isolated by collagenase digestion as described by Walker et al. [51]. Cells were cultivated in VascuLife^TM^ EnGS cell culture medium (Lifeline Cell Technology, Walkersville, MD, USA), a basal medium with a supplemental kit: 0.2% EnGS, 5 ng/mL rh epidermal growth factor (EGF), 50 μg/mL Ascorbic Acid, 10 mM l-Glutamine, 1 μg/mL Hydrocortisone Hemisuccinate, 0.75 Units/mL Heparin Sulfate, 2% FBS, 100 U/mL Penicillin, 100 μg/mL Streptomycin, and 10 ng/mL Amphotericin. Passages from four to seven were used for experiments.

### 4.7. Measurement of pH Value

PLGA degrades into lactic acid and glycolic acid by hydrolysis, whereby pH value shift is possible. Thus, environment for cells could change and influence them negatively in cell proliferation right up to cell death. Therefore, coverslips (15 mm diameter) were coated with 50 µg of three different PLGA solutions: (1) RG 752 H 75:25, (2) PLGA 85:15, and (3) RG 756 S 75:25. Slides were air-dried overnight and incubated 2 mL in medium each slide under standard cultivation conditions. Uncoated glass slides served as a control. Medium was measured at time zero which served as a baseline. The first pH value was determined by measuring the supernatant of each sample after one week and continued for four weeks.

### 4.8. PLGA/RNA Mediated Transfection of EA.hy926 and hVECs

First, 100,000 EA.hy926 or 120,000 hVECs were seeded one day before in 24-well plates. For co-transfection approach 130,000 EA.hy926 were seeded in one well of a 12-well plate. We decided us to use this amount of cells to reach 80% confluency on the next day. Previous experiments showed that the highest transfection efficiencies are achieved with this amount of cells. PLGA coatings were dried overnight and glass slides or coverslips were laid down with the coated side on the medium refreshed cell layer. This so called reverse assay was first described by Wintermantel et al. and applied in our laboratory for many substrate-mediated transfection tests [52]. Transfection time of EA.hy926 mediated by PLGA/RNA coatings was: 24 h for transfection efficiency and 48 h for (a) knockdown experiments, (b) immune response tests (plus hVECs), and (c) 5′-RNA ligase mediated rapid amplification of cDNA-ends PCR assay. The incubation time for knockdown evaluation was reduced to 24 h for hVECs in knockdown experiments due to their sensitivity. After transfection, cells were prepared for flow cytometry or quantitative Real-Time PCR (qRT-PCR). In the long-term transfection efficiency approach, the coated glass slides were laid down on always newly prepared EA.hy926 for 24 h until day 10. At later time points, the cells were incubated for 48 h with the slides. Herein, we always used the initially coated glass slides from day one to the last day. If cells were cultured for 48 h, medium was replaced every 24 h. After 24 h (days 1–10) or 48 h (days 12–20), cells were analyzed by flow cytometry. For co-transfection of mRNA and siRNA, cells were transfected for 48 h and subsequently analyzed by flow cytometry or fluorescence microscopy.

### 4.9. Cell Viability Assay

The three different polymers were tested for cell compatibility by MTT-assay and by CASY^®^ cell counter (Schärfe System, Reutlingen, Germany). For both tests, coverslips were coated as mentioned before (measurement of pH-value). Coated slides were incubated in medium for one, two, three, and four weeks at standard culture conditions and supernatants were used for cell cultivation. Then 100,000 EA.hy926 were seeded in a 24-well plate and cultivated for 48 h with the obtained supernatants. Then, cells were detached for cell count measurement with CASY^®^ cell counter, which detects living cells by an electronic pulse area analysis. For the MTT-assay, used medium was removed and replaced by 300 µL RPMI without phenol red and supplemented with 10% MTT (5 mg/mL). After 4 h incubation, MTT solution was aspirated and 200 µL dimethyl-sulfoxide was added to each well and gently shaked. Absorbance was measured at wavelength 540 nm by a microplate reader (Mithras LB 940, Berthold Technologies, Bad Wildbad, Germany).

### 4.10. Hemocompatibility Testing

Medical devices must be verified in respect of biocompatibility which is separated in two fields: cytocompatibility and hemocompatibility. The EN ISO 10993–4 demands for at least one test addressing thrombosis/coagulation, hematology, inflammation, and complement system to determine the compatibility of a medical device in combination with blood.

Hemocompatibility of PLGA 1–3 was tested with polymer-coated coverslips as described in measurement of pH-value. Coverslips were incubated in 12-well plates at 37 °C under gentle shaking with 3 mL human blood from six independent blood donors. After 1 h incubation, blood was pooled for blood cells analysis like leukocytes, erythrocytes, and platelets. A Micros 60 counter (ABX Diagnostics, Montpellier, France) counted the blood cell numbers. Protein expression of C3a and SC5b9 which were associated with the complement system was analyzed by ELISAs (both Osteomedical GmbH, Bünde, Germany). Additionally, polymorphonuclear-Elastase (PMN-elastase) was tested as a sign of degranulation of leukocytes during an inflammatory reaction (Demeditec Diagnostics, Kiel, Germany). β-Thromboglobulin expression, associated with activated platelets, was determined by ASSERA-CHROM^®^ β-TG kit (Diagnostica Stago, Asnieres, France). The measurement of thrombin-antithrombin complex (TAT) with ELISA based Enzygnost^®^ TAT micro enzyme immunoassay (Dade Behring, Marburg, Germany) gives information about the alteration of coagulation activity.

### 4.11. Flow Cytometry

Transfection efficiency or knockdown of PLGA/RNA coatings was determined by flow cytometry after 24 h or 48 h, respectively. Glass slides were removed after incubation time and cells were washed, detached and fixed with 2.5% paraformaldehyde (PFA). For knockdown experiments, cells were additionally stimulated with 5 ng/mL tumor necrosis factor (TNF)-α (BD Biosciences, Germany) for 12 h to induce ICAM-1 expression after the washing step. Immunofluorescence staining was prepared with PE mouse anti-human CD54, diluted in a 0.5% FBS/PBS solution, (BD Bioscience, Germany) for 30 min at 37 °C with following detachment and fixation of the cells as stated above. Flowcytometric analysis was performed with 10,000 cells/measurement (FACScan™, Becton Dickinson GmbH, Heidelberg, Germany) and evaluated with CellQuestPro software (version 4, Becton Dickinson GmbH). The geometric mean served for evaluating the results.

### 4.12. Quantitative Real-Time PCR (qRT-PCR)

First of all, total RNA from PLGA/RNA transfected EA.hy926 and hVECs was isolated using Aurum™ total RNA mini kit (Bio-Rad Laboratories, Inc., Hercules, CA, USA). RNA was quantified and 200 ng of each RNA sample was utilized for the iScript™ cDNA Synthesis Kit from (Bio-Rad) according to the manufacturer’s instructions for reverse transcription. Primer design was done with NCBI primer-blast, the primer sequences in Table 2, synthesized by Operon (Köln, Germany), were used for qRT-PCR. Poly (IC) dsRNA from R&D systems (Minneapolis, MN, USA) served as positive control for immune stimulation. PCR mixes contained IQ™SYBR^®^Green Supermix (Bio-Rad), 400 nM forward and reverse primer, and 2 ng of cDNA in a total volume of 15 µL. All samples were performed in triplicates. Normalized gene expression was calculated by the threshold cycle (ΔCt) method using GAPDH as a reference.

### 4.13. 5′-RNA Ligase–Mediated Rapid Amplification of cDNA-Ends PCR (5′-RLM-RACE-PCR)

**Figure 9:**
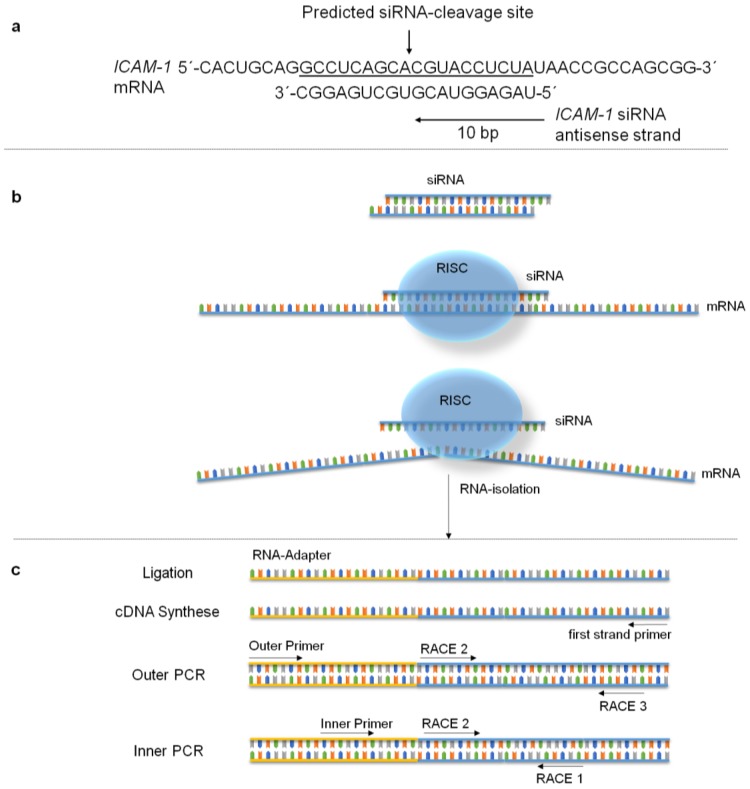
Illustration of the RNA Interference mechanism and the 5´-RLM-RACE PCR technique. (**a**) Shows the predicted siRNA cleavage site in the ICAM-1 mRNA. (**b**) Illustrates the RNAi mechanism in the cytoplasm of EA.hy926 cells. (**c**) Demonstrates the 5´-RLM RACE PCR technique, starting with total RNA Isolation, then the RNA adapter is ligated to cleaved mRNA strands. Afterwards RNA is reverse transcribed gene-specifically, with specific first-strand synthesis primers for ICAM-1. Subsequently, Outer PCR and Inner PCR with gene-specific primers (RACE 1, 2, and 3) and adapter-specific primers (Outer and Inner Primer) are conducted. Only if siRNA-mediated cleavage occurs specific products are produced with primer combinations of Inner Primer and RACE 1. These PCR products are then sequenced to prove siRNA-specific degradation.

This technique which was established to detect siRNA-mediated mRNA cleavage was first described by Soutschek et al. in 2004 [53]. Later Davis et al. used this technique to verify siRNA-mediated mRNA cleavage in a human phase I clinical trial [54]. This technique detects cleaved mRNA and is especially suitable to confirm the mRNA degradation dependent on the base pair sequence of the siRNA in transfection assays. Therefore, 50 µg of PLGA and 3 µg ICAM-siRNA were dried on glass slides, the glass slides were laid down on confluent EA.hy926 cells for 48 h and cells were stimulated for 12 h with the above stated TNF-α concentration. Subsequently, the RNA was isolated as described above. For our study we used components of the commercially available First Choice^®^ RLM-RACE Kit from Life technologies (Darmstadt, Germany). The kit is delivered with RNA-Adapter and T4 RNA Ligase to conduct the 5′ RACE Adapter Ligation. We used 2 µg of isolated RNA for ligation. Also the kit contains M-MLV Reverse Transcriptase for first strand cDNA synthesis where we used 200 ng of ligated RNA. The user has to design gene-specific primers for the first strand synthesis and also for the PCR of the Outer and Inner PCR which are following after first strand cDNA synthesis. Primer design was done again with the software ‘Primer3’ [55] and Primer Premier 5 (PREMIER Biosoft International). Primer sequences that were used for 5-RLM-RACE-PCR are shown in Table 1. All PCR reactions (Outer and Inner PCR) were conducted as a qRT-PCR and contained IQ^TM^SYBR^®^Green Supermix from Bio-Rad (Hercules, CA, USA), 400 nM forward and reverse primer and 2 ng of cDNA in a total volume of 15 µL. All PCR reactions were performed in triplicates. For better understanding of this technique we also designed an illustration (Figure 9).

### 4.14. Statistics

All experiments described in this work were done at least three times independently. Values showing variation were analyzed by an outlier test with a significance value of 0.05. The comparison of different samples was done by ANOVA and Bonferroni correction as a Post-test.

## 5. Conclusions

PLGA seems to be an excellent biodegradable and hemocompatible RNA NP delivery carrier, especially regarding the challenge for innovative improvements in DES. In combination with the powerful mechanism of RNAi, the gene silencing of undesired CAMs during inflammation could be achieved. All three PLGA resomers were non-toxic to EA.hy926 cells and proved hemocompatible with no adherence of blood cells on the PLGA coatings, a crucial point with respect to preventing thrombosis after stent implantation. The siRNA NPs incorporated in the PLGA 1–3 coatings were released and able to transfect EA.hy926 cells, where PLGA 1 with 3 µg siRNA was the best combination with 95% transfection efficiency. Furthermore, siICAM-1 NPs in PLGA 1 provoked the highest ICAM-1 knockdown of 36%. These findings and the results of pH stability indicated that PLGA 1 is degraded the fastest. With our novel multilayer build-up of PLGA 1 and siRNA NPs, we demonstrated the co-transfection of eGFPmRNA and siRNA. Furthermore, our multilayer coating with siRNA has the potential to transfect cells with high efficiency in the first two days, with a burst release until day six, and with a sustained slow release thereafter. Considering our results, we finally summarize that the delivery carrier PLGA combined with siICAM-1 NPs has great potential for the development of new a era of stents preventing ISR.

## Figures and Tables

**Table 1:** Transfection efficiency values of co-transfection with eGFPmRNA and siRNA AF 555. (++): without Lipofectamine^®^ 2000 and PLGA 1; (+): without Lipofectamine^®^ 2000; *: without; + with. Ctrl = control.

Positive Cells (%)	Ctrl (++)	*mRNA* siRNA (+)	*mRNA* siRNA	*-mRNA +siRNA	+mRNA *siRNA	+mRNA +siRNA
siRNA positive	0.03	0.05	0.08	26.03	0.01	6.25
mRNA positive	0.94	2.19	2.86	0.26	10.91	7.90
siRNA + mRNA positive	1.02	1.72	3.18	4.33	2.84	11.01

**Table 2:** Primer sequences for qRT-PCR and primer sequences for 5′-RLM-RACE PCR purchased from MWG Operon (Ebersberg, Germany).

**qRT-PCR Primer**	**Sequence**
OAS	forward: 5’-GGAGACAGCTGGAAGCCTGTC-3’ reverse: 5’-TGACCCAGGGCATCAAAGG-3’
STAT-1	forward: 5’-TGGAAGCGGAGACAGCAGAG-3’ reverse: 5’-AGGTGTATTTCTGTTCCAATTCCTC-3’
CXCL-10	forward: 5’-AAGTGGCATTCAAGGAGTACC-3’ reverse: 5’-ACGTGGACAAAATTGGCTTGC-3’
ICAM-1	forward: 5’-CTTGAGGGCACCTACCTCTGTC-3’ reverse: 5’-CGGCTGCTACCACAGTGATG-3’
ß-Actin	forward: 5’-GAGCACAGAGCCTCGCCTTT-3’ reverse: 5’-TCATCATCCATGGTGAGCTGG-3’
**5′-RLM-RACE PCR**	**sequence**
ICAM-1 first strand	5´-AGGTACCATGGCCCCAAATG-3´
ICAM-1 RACE 1	5´-ACTCTGTTCAGTGTGGCACC-3´
ICAM-1 RACE 2	5´-TCTTCCTCGGCCTTCCCATA-3´
ICAM-1 RACE 3	5´-TGGCCCCAAATGCTGTTGTA-3´
RNA-Adapter (universally)	5´-GCUGAUGGCGAUGAAUGAACACUGCGUUUGCUGGCUUUGAUGAAA-3´
Outer Primer (universally)	5´-GCTGATGGCGATGAATGAACACTG-3´
Inner Primer (universally)	5´-CGCGGATCCGAACACTGCGTTTGCTGGCTTTGATG-3´

## Supplementary Materials

The following are available online at http://www.mdpi.com/1424-8247/10/1/23/s1, Figure S1: Relative viability of EA.hy926 cells analyzed by CASY after incubation with supernatant of incubated PLGA 1–, 2– or 3–coverslips; Figure S2: Expression of ICAM-1 mRNA after 48 h transfection by PLGA siICAM or SCR-siRNA–coated slides, quantified by qRT-PCR; Figure S3: Fluorescence microscopy of cells co-transfected with AF555-siRNA and eGFP-mRNA.
