# Supplementary material for: RNA-Eluting Surfaces for the Modulation of Gene Expression as A Novel Stent Concept
Source: Pharmaceuticals (Basel). 2017 Feb 10;10(1):23. doi: 10.3390/ph10010023 (PMC5374427; doi:10.3390/ph10010023)
Supplement: Supplementary file 1 [file pharmaceuticals-10-00023-s001.pdf]

Supplementary Materials: RNA-Eluting Surfaces for the Modulation of Gene Expression as A Novel Stent Concept

Olivia Koenig, Diane Zengerle, Nadja Perle, Susanne Hossfeld, Bernd Neumann, Andreas Behring, Meltem Avci-Adali, Tobias Walker, Christian Schlensak, Hans Peter Wendel and Andrea Nolte

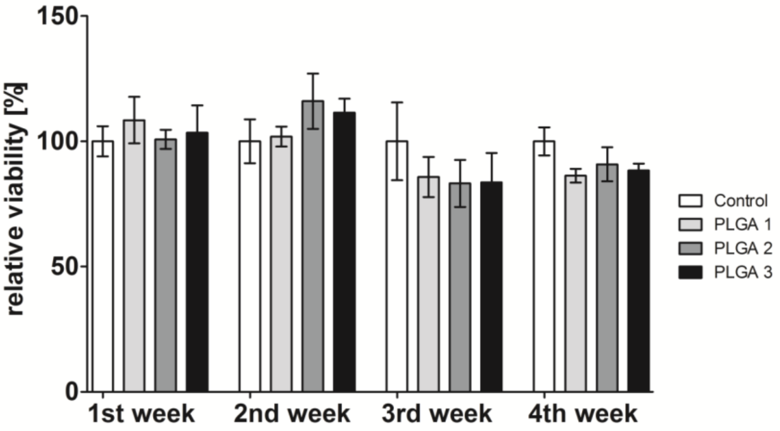

Figure S1. Relative viability of EA.hy926 cells analyzed by CASY after incubation with **s**upernatant of incubated PLGA 1, 2 or 3 coated coverslips. Therefore, 100,000 EA.hy926 cells were seeded in one well of a 24-well plate and cultivated with the supernatant of the respective cultivated PLGA slides for 48 h. For the control group, medium was incubated with coverslips and supernatant added to the cells. The control was set to 100%, each bar represents the mean ± standard error (SEM) of $n = 1$.

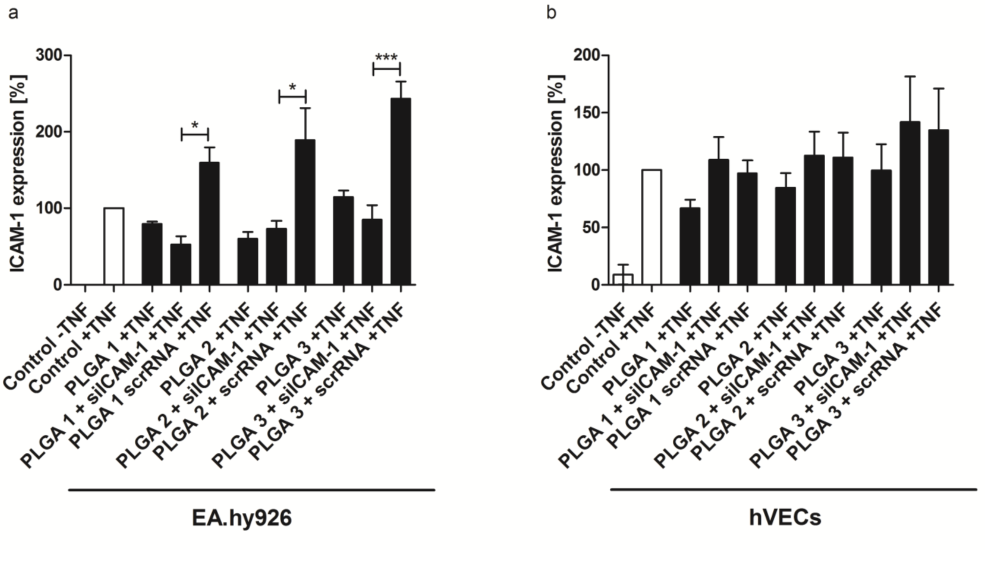

Figure S2. Expression of ICAM-1 mRNA after 48 h transfection by PLGA siICAM or SCRsiRNA coated slides, quantified by qRT-PCR. (**a**) ICAM-1 expression in EA.hy926, (**b**) ICAM-1 expression in hVECs. PLGA 1–3 coated glass slides were placed onto cell layer for 48 h. Each bar represents the mean ± standard error of $n = 3$ with EA.hy926 and $n = 5$ with hVECs. * Statistical significance $p < 0.05$; *** statistical significance $p < 0.001$.

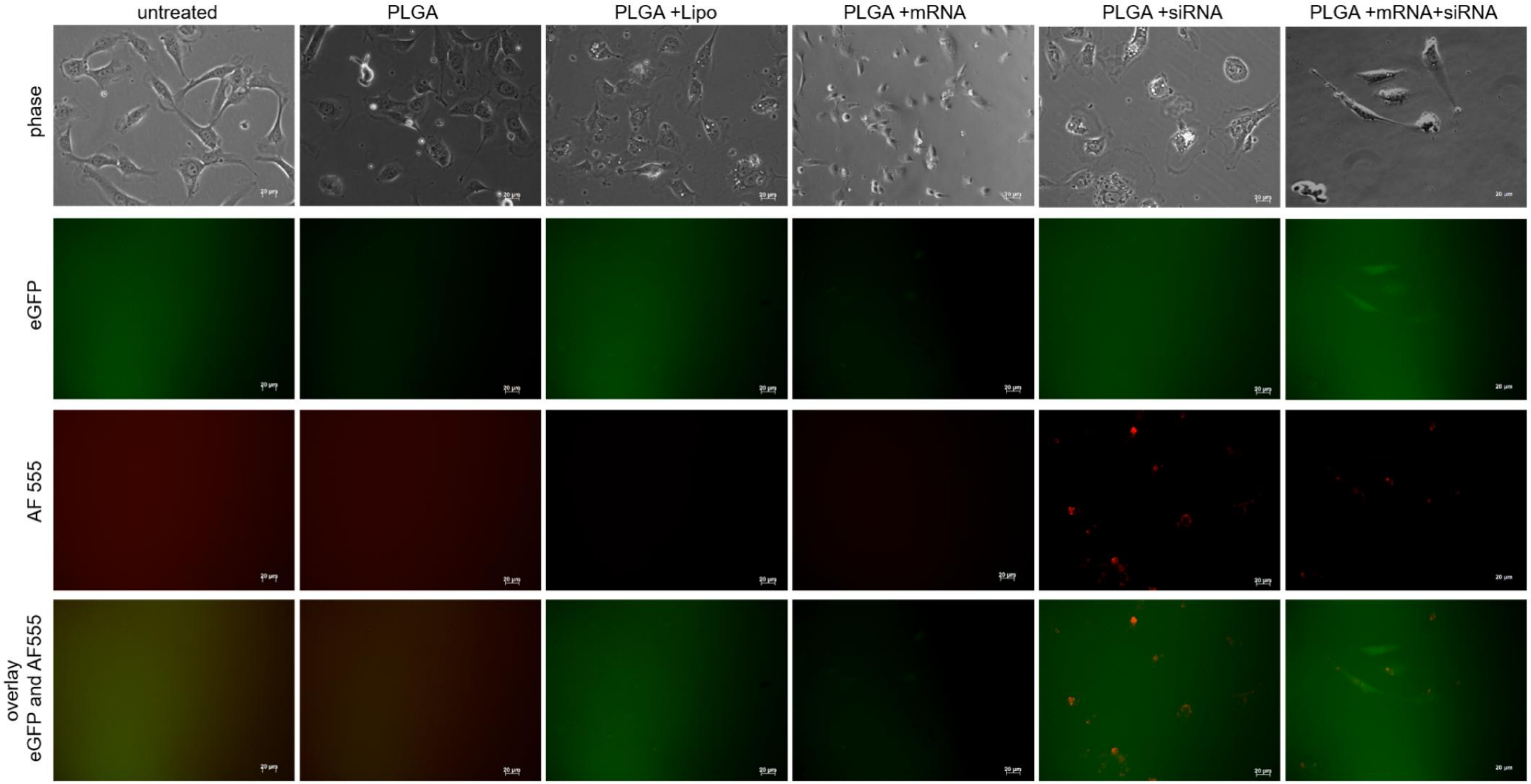

Figure S3. Fluorescence microscopy of cells co-transfected with AF555-siRNA and eGFP-mRNA. The following samples were analyzed: untreated cells, PLGA without RNAs or transfection reagent (+PLGA, −mRNA, −siRNA), PLGA with Lipofectamine® 2000 (+PLGA, +Lipo, −mRNA, −siRNA), PLGA with Lipofectamine® 2000 and mRNA (+PLGA, +Lipo, −siRNA, +mRNA), PLGA with Lipofectamine and siRNA (+PLGA, +Lipo, +siRNA, −mRNA), and PLGA with Lipofectamine, siRNA and mRNA (+PLGA, +Lipo, +siRNA, +mRNA).
